# Seaweed-Liked WS_2_/rGO Enabling Ultralong Cycling Life and Enhanced Rate Capability for Lithium-Ion Batteries

**DOI:** 10.3390/nano9030469

**Published:** 2019-03-20

**Authors:** Yi Huang, Yu Jiang, Zhaofei Ma, Yan Zhang, Xianfeng Zheng, Xuemin Yan, Xiaoqing Deng, Wei Xiao, Haolin Tang

**Affiliations:** 1 College of Chemistry and Environmental Engineering, Yangtze University, Jingzhou 434023, China; h-uangy-i@163.com (Y.H.); librajiangyu@163.com (Y.J.); libramzf@163.com (Z.M.); YanZhang@126.com (Y.Z.); XianfengZheng@126.com (X.Z.); XiaoqingDeng@126.com (X.D.); WeiXiao@126.com (W.X.); 2 State Key Laboratory of Advanced Technology for Materials Synthesis and Processing, Wuhan University of Technology, Wuhan 430070, China

**Keywords:** WS_2_/rGO composites, seaweed-liked structure, anode, ultralong cycling life, Lithium ion batteries

## Abstract

WS_2_ is considered as a potential anode material for lithium ion batteries (LIBs) with superior theoretical capacity and stable structure with two-dimensional which facilitates to the transportation and storage of lithium ion. Nevertheless, the commercial recognition of WS_2_ has been impeded by the intrinsic properties of WS_2_, including poor electrical conductivity and large volume expansion. Herein, a seaweed-liked WS_2_/reduced graphene oxide (rGO) composites has been fabricated through a procedure involving the self-assembling of WO_4_^2−^, hexadecyl trimethyl ammonium ion with graphene oxide (GO) and the subsequent thermal treatment. The WS_2_/rGO nanocomposite exhibited the outstanding electrochemical property with a stable and remarkable capacity (507.7 mAh·g^−1^) at 1.0 A·g^−1^ even after 1000 cycles. This advanced electrochemical property is due to its seaweed-liked feature which can bring in plentiful active sites, ameliorate the stresses arisen from volume variations and increase charge transfer rate.

## 1. Introduction

Lithium ion battery, as a green energy storage device, is the alternative to current batteries most potential (for example, shows advanced energy density, which is about 4 times higher than nickel-cadmium battery and 1.6 times higher than nickel-metal hydride battery) [1,2,3]. However, the ever-lasting need for powerful energy storage equipment, especially for emerging industry (such as electric vehicle and smart grid), has spurred the evolution of the electrode material for LIBs [4,5,6,7]. Recently, tremendous researches are focus on transition metal oxides (TMOs) attributing to their highly specific capacity (almost 3 or 4 times higher than graphite). Nonetheless, the practical application of TMOs are hampered by their intrinsic property (e.g., poor ion conductivity, and low structural stability origin from the conversion reaction mechanism). To address these issues, two-dimensional (2D) inorganic transition metal dichalcogenides (TMDs) have been introduced, ascribing to their relative high electronic conductivity, superior specific capacity, marvelous structural stability and environment benignity [8,9,10,11,12,13,14,15].

Tungsten disulfide (WS_2_), as an important component of the TMDs, has drawn extensive attention owing to its superior theoretical capacity and abundant reserves [16,17,18,19,20]. WS_2_ is a hexagonal crystal system, and the underlying crystal structure of WS_2_ is composed of three atomic layers connected by van der Waals forces, stacked in the manner of S–W–S, providing the channel for Li^+^ ions to be embedded and released [21,22,23,24,25]. Moreover, the large spacing of the WS_2_ adjacent layers is more favorable to the transport and the accumulation of lithium ions, which leads to the fact that the geometric structure of the WS_2_ can be preserved without an apparent variation during the corresponding electrochemical reaction [26,27,28]. However, the large-scale commercial application of WS_2_ has been impeded by the intrinsic properties of WS_2_, including weak electrical conductivity and volume expansion [12,29,30]. The intrinsic properties can cause the collapse of geometrical configuration in cycling processes, followed by a dramatic performance reduction. In this case, enhancing the conductivity of WS_2_ and improving structural stability are a potential approach to overcome these obstacles. Several reports have showed that combining WS_2_ with the conductive matrix is a promising means to enhance the electrochemical properties, as well as a potential way to alleviate the large volume expansion of the active material [28,31,32,33]. Graphene is a perfect compound material with advanced electrochemical characteristics and mechanical strength [34]. Li et al. successfully fabricated multi-slices WS_2_/rGO nanocomposites by hydrothermal synthesis method [35]. The composite could exhibit a capacity of ~337 mAh·g^−1^ at 2.0 A·g^−1^ after 100 cycles. In addition, multitudinous other various conductive matrix materials have been applied to address these limitations. Ren et al. reported the foam structure WS_2_/single-wall carbon nanotube nanocomposites delivers a reversible capacity (~688 mAh·g^−1^) for 1000 cycles at 0.1 A·g^−1^ [36]. Wu et al. designed WS_2_/carbon nanofiber composites via combining hydrothermal and electrospinning methods with the presence of a capacity of 545 mAh·g^−1^ at 0.5 A·g^−1^ [37], while Kong et al. prepared WS_2_/graphitic carbon nanotubes through combining electrospinning and chemical vapor deposition (CVD) with 570 mAh·g^−1^ at 0.2 A·g^−1^ [38]. Unfortunately, a variety of WS_2_ nanocomposites above have been synthesized for LIBs, either the preparation method is too complicated for large-scale production, or the long preparation period increases the production cost, or the cycling performance is slightly insufficient, hindering their further commercial application.

Herein, for higher energy density and a high-rate capability, we synthesized a seaweed-liked WS_2_/rGO nanocomposites through a procedure involving the self-assembling of WO_4_^2−^, hexadecyl trimethyl ammonium ion with GO and the subsequent thermal treatment. The seaweed-liked WS_2_/rGO nanocomposites, as an anode material, displays an ultralong cycling life and striking rate performance, it can provide a prominent reversible capacity of 507.7 mAh·g^−1^ after 1000 cycles at 1.0 A·g^−1^ and a specific capacity of 108 mAh·g^−1^ at 20.0 A·g^−1^, which shows a highly attractive as the electrode to next-generation LIBs.

## 2. Experimental

### 2.1. Preparation of Graphene Oxide (GO)

GO was prepared from flake graphite (325 meshes) via a modified Hummers’ method. Under the condition of ice bath, NaNO_3_ (2.5 g) and flake graphite (2.0 g) were added to 180 mL of concentrated sulphuric acid (H_2_SO_4_, 95%) while stirring. Subsequently, KMnO_4_ (15.0 g) was slowly added, and the temperature of the reaction in the process was controlled below 20 °C. Then, 180 mL of DI water was introduced after reaction for 24 h. Next, raise the temperature to 98 °C and maintain for ~1 h. When the temperature dropped to 70 °C, 80 mL of hydrogen peroxide aqueous solution (H_2_O_2_, 35%) was added. Continue to stir for 1 h after cooling to room temperature. Ultimately, the acquired GO was centrifuged several times with 5% dilute hydrochloric acid and DI water, and then freeze-dried for preservation. The resistance of the deionized (DI) water used for the reaction was ~18 MΩ cm^−1^.

### 2.2. Synthesis of WS_2_/rGO Composites

To fabricate WS_2_/rGO composite, GO (40 mg), cetyltrimethyl ammonium bromide (CTAB) (0.364 g), ammonium tungstate (0.5 g) and thiourea (1.83 g) were subsequently dispersed in 25 mL DI water with violent stirring. After freeze-dried, the resulting mixture was heated to 500 °C and kept in an Ar flowing tube furnace for 3 h. The synthesis approach for bare WS_2_ were the same as the above composite without the addition of GO.

### 2.3. Characterization Measurements

The obtained samples were analyzed by the scanning electron microscope (SEM, Hitachi S5500, Hitachi, Tokyo, Japan); the field emission transmission electron microscope (FETEM, JEM-2100F, JEOL, Tokyo, Japan); the X-ray photoelectron spectroscopy (XPS, Thermo Escalab 250, Thermo Fisher Scientific, Waltham, United States) with a focused monochromatic Al Kα X-ray source (1486 eV); X-ray diffraction (XRD) patterns (Rigaku MiniFlex 600 X-ray diffractometer, Rigaku, Tokyo, Japan) with Cu Kα (λKα = 1.5406 Å) as the radiation source, and laser confocal Raman microspectroscopy (Raman, Horiba Jobin-Yvon LabRAM HR800, Horiba Jobin-Yvon, Paris, France). The electrochemical impedance spectroscopy (EIS) were measured on a CHI660E (CH instrument, Shanghai, China) in the frequency range of 100 kHz to 0.01 Hz, with an amplitude of 10 mV.

### 2.4. Electrochemical Measurements

The resulting sample is assembled into the CR 2025 coin-type half cells in a glove box at Ar atmosphere for electrochemical performance testing. 1 M LiPF_6_ solution (EC/EMC/DEC = 1:1:1 *v*/*v*) as the electrolyte, Celgard 2400 membrane as the separator and the lithium metal as the counter/reference electrode. The working electrodes were made of the resulting sample (80 wt%), acetylene black (10 wt%) and polyvinylidene fluoride (10 wt%) dissolved in N-methyl-2-pyrrolidinone to form slurry. The obtained homogeneous paste were pasted on Cu foils with a thickness of ~20 µm and dried in vacuum oven at 90 °C for 12 h and then cut into a wafer with a diameter of 12 mm, each wafer possesses about 1.9 mg active material. The cyclic voltammetry (CV) (CHI660E electrochemical workstation) and galvanostatic charge/discharge cycles (Arbin battery test system, current rate = (0.1 − 20.0 A·g^−1^)) were tested within the 3.0–0.01 V voltage range. The electrochemical impedance spectroscopy (EIS) were measured on a CHI660E in the frequency range of 10^5^ Hz to 10^−2^ Hz, with an amplitude of 10 mV.

## 3. Results and Discussion

**Figure 1:**
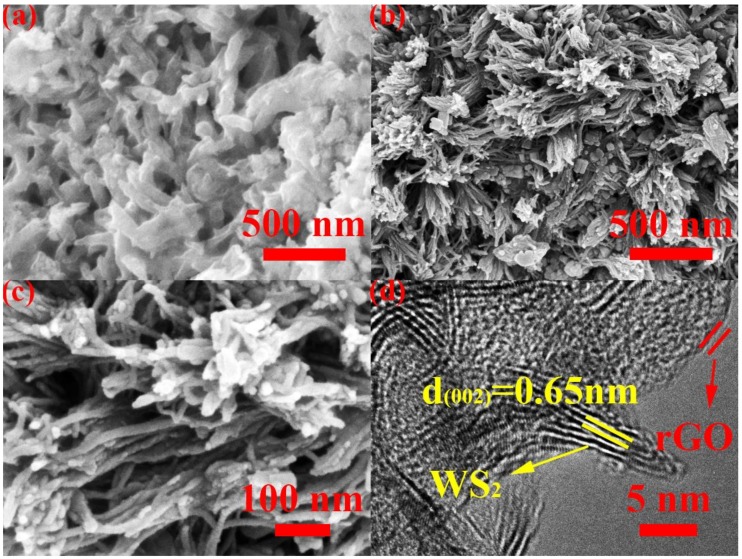
(**a**) SEM images of bare WS_2_ and (**b**–**c**) SEM, (**d**) TEM images of WS_2_/rGO composites.

**Figure 2:**
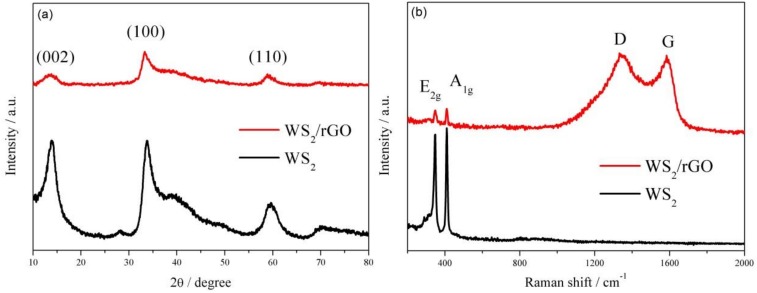
(**a**) XRD patterns and (**b**) Raman spectra of WS_2_ and WS_2_/rGO.

The morphological characteristics of the two prepared nanomaterials were researched by observing the images of SEM and TEM (Figure 1). The WS_2_/rGO nanocomposites was fabricated with a procedure involving the self-assembling of WO_4_^2−^, CTA^+^ with GO and the subsequent thermal treatment. The obtained WS_2_/rGO sample exhibits a uniform seaweed-liked morphology in Figure 1b. There is no sight of nanosheets from rGO. Since CTA^+^ facilitates to reduce the innate charge incompatibility between negatively charged GO and WO_4_^2−^, ascribing to the electrostatic interaction, there is no conspicuous interfacial structure between rGO and WS_2_ (synthesis from the subsequent calcination with the presence of thiourea) in Figure 1c. The high magnification of WS_2_/rGO can be seen in Figure 1c. In contrast, the bare WS_2_ without GO just form short rod shape in Figure 1a, which illustrates that GO plays an indispensable role in the production of the seaweed-liked nanostructures. To further investigate the structure of WS_2_/rGO nanocomposite, Figure 1d displays the TEM images of WS_2_/rGO nanocomposite. The spacing of the clear lattice fringe is 0.65 nm, corresponding to the d-spacing of (002) plane of the c-axis of hexagonal WS_2_. The thin rGO is attached to the surface of the few layer WS_2_ nanosheets as shown in Figure 1d. The added GO is reduced to rGO during the calcining phase and formed stable seaweed-liked composite with WS_2_, not only providing an efficient conductive path for electron transport, but also avoiding a structure collapse of the electrode, owing to the huge volume expansion. Which is consistent with the XRD patterns shown in Figure 2a.

Figure 2a shows X-ray diffraction patterns of the two samples. All conspicuous diffraction peaks (at 2θ = 13.52°, 33.81°, 59.28°) can be easily linked to the 2H-WS_2_ without the existence of other phases or impurities, demonstrating the high purity of the obtained WS_2_ (Figure 2a). Furthermore, a few-layered structure of WS_2_ can be certified by the presence of (002), (100) and (110) reflections. Since the existence of the rGO diminishes the intensity of incoming and reflected X-ray light, the reduction of the diffraction peaks intensity of WS_2_/rGO can be clearly observed. The similar attenuation also can be observed in Raman spectra (Figure 2b), where the intensity of the characteristic Raman signature of WS_2_ (located at 346 and 409 cm^−1^ correspond to the E_2g_ and A_1g_ modes, respectively) in WS_2_/rGO shows obviously weaker than that of bare WS_2_. Moreover, Raman spectrum of WS_2_/rGO shown in Figure 2b also can certify the existence of rGO attributing the emergence of two prominent peaks D (relate to the symmetric k-point phonon pattern of A_1g_) and G (ascribing to the E_2g_ phonon of C sp^2^ atoms).

**Figure 3:**
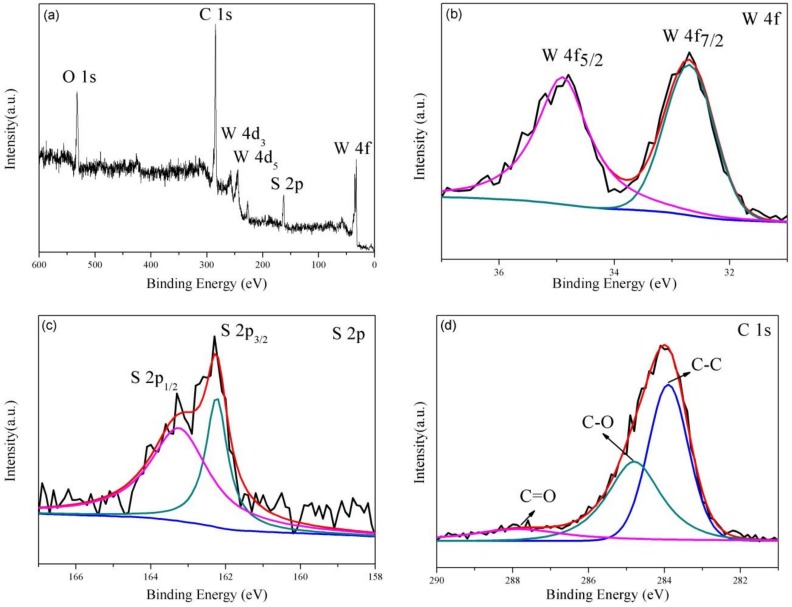
(**a**) broad XPS scan spectrum of WS_2_/rGO composites; the high-resolution spectrum of (**b**) W 4f; (**c**) S 2p; (**d**) C 1s.

XPS analyses were applied to identify the chemical composition of the WS_2_/rGO sample (Figure 3). The presence of W, S, C and O (originate from the unreduced oxygeneous groups of the rGO) in the WS_2_/rGO composite is certified by survey spectrum shown in Figure 3a. The distribution of the W 4f peaks on WS_2_ can be testified through two obviously peaks at 34.9 and 32.7 eV (in Figure 3b), corresponding to the W 4f_5/2_ and W 4f_7/2_ spin peaks of WS_2_, respectively. Meanwhile, the high-resolution spectrum of S (Figure 3c) illustrates two characteristic peaks at 162.0 and 163.0 eV that stemmed from the S^2−^ typical of WS_2_. The high-resolution spectral of C 1s is presented in Figure 3d, where three different components (in 287.8, 284.8 and 284.1 eV) can be observed, corresponding to the C=O, C–O and C–C bonds of rGO, respectively, which in line with the previously reports [39,40,41].

**Figure 4:**
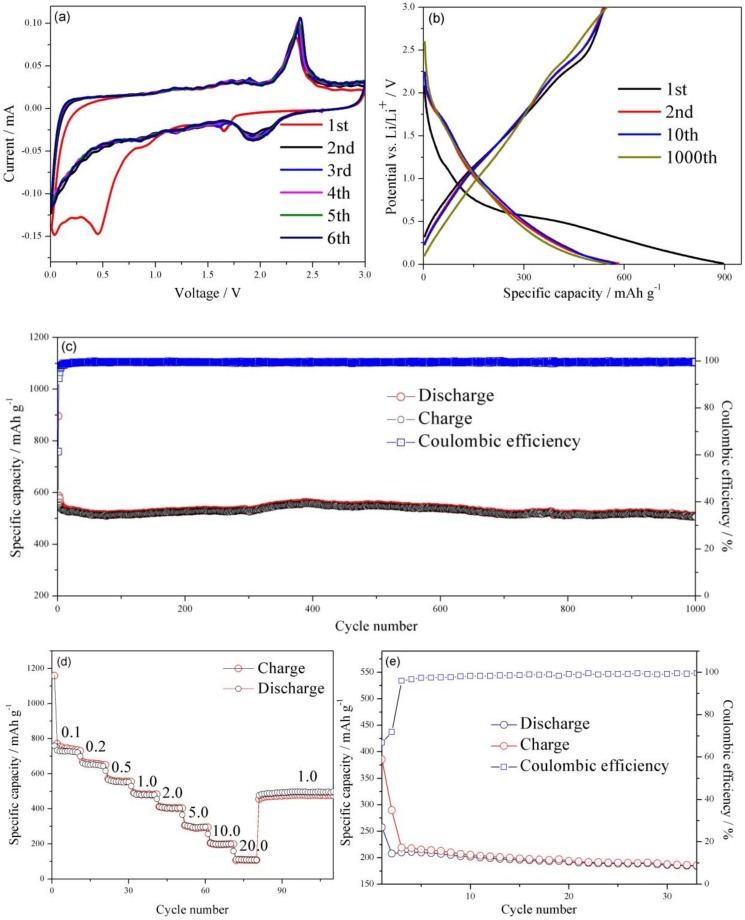
(**a**) CV curves of the WS_2_/rGO composite at 0.01 mV·s^−1^ in the 0.01 to 3.0 V voltage window. (**b**) Charge/discharge voltage profiles of WS_2_/rGO at 1.0 A·g^−1^. (**c**) The cycle stabilities of the WS_2_/rGO at 1.0 A·g^−1^. (**d**) The rate capacities of WS_2_/rGO at 0.1, 0.2, 0.5, 1.0, 2.0, 5.0, 10.0 and 20.0 A·g^−1^. (**e**) The cycle stabilities of the bare WS_2_ at 1.0 A·g^−1^.

The electrochemical process of the WS_2_/rGO composite electrode in charge-discharge cycling was investigated by the initial six cycles cyclic voltammogram (CV) at 0.01 mV·s^−1^ in Figure 4a. As shown in Figure 4a, the reduction peak at 1.65 V and oxidation peak at 2.35 V can be observed clearly in the first cycle, which was caused by the insertion/extraction reaction of Li^+^ in the WS_2_ interlayer space (WS_2_ + xLi^+^ + xe^−^ ↔ Li_x_WS_2_). Another obvious reduction peak can be seen at 0.45 V that arises from the reduction of WS_2_ (WS_2_ + 4Li^+^ + 4e^−^ → W + 2Li_2_S), and accompanying by non-aqueous electrolyte decomposition and adverse reactions between Li^+^ and the residual oxygen-containing functional groups. In the second cycle onwards, reduction peaks at 1.65 and 0.45 V no longer can be observed, meanwhile, a new reaction at 1.75–2.18 V can be visualized. This change could be accounted by the production of gel-liked solid electrolyte interphase (SEI) film [42]. Moreover, those cyclic voltammograms were nearly fully superimposable, indicating the excellent reversibility and cycle stability of the WS_2_/rGO nanocomposites. 

Figure 4b illustrates the charge-discharge curves of WS_2_/rGO material at 1st, 2nd, 100th and 1000th cycles under 1.0 A·g^−1^, respectively. The voltage plateau appears in the first discharge curve around 0.55 V, which may be ascribed to WS_2_ being reduced to metallic W at the same time that Li_2_O is generated. The first discharge/charge capacity of the WS_2_/rGO composites comes to 895.8 mAh·g^−1^ and 546.3 mAh·g^−1^, causing a high premier Coulombic efficiency of 60.9%. The nonreversible loss of the first cycle is probably ascribed to the adverse reactions between Li^+^ and the residual oxygen-containing functional groups on the WS_2_/rGO composites, and the formation of SEI film during lithium ion intercalation process [43,44,45,46,47], which makes the following discharge curves exhibit a totally different voltage plateau at 2.0 V, in conformity to the aforementioned CV curves, suggesting the superior electrochemical property of the WS_2_/rGO composites. This was also explained by the similarity between the 2nd, the 10th and the 1000th charge/discharge profiles of the WS_2_/rGO (Figure 4b) and their near coulombic efficiency after the first several cycles in Figure 4c. Furthermore, the WS_2_/rGO composites electrodes present a marvelous cycle stability at 1.0 A·g^−1^ (Figure 4c). Even after 1000th cycle, the WS_2_/rGO composites electrode still have a charge capacities of 505.6 mAh·g^−1^ and a discharge capacities of 507.7 mAh·g^−1^. Compared with the capacities of another WS_2_-based anode materials report previously, the stable reversible capacity of the WS_2_/rGO composites is much higher, suggesting the superiority of using the WS_2_/rGO composites as an anode material for LIBs [12].

To further study the advanced electrochemical characteristics of the WS_2_/rGO composites electrode, rate capability also has been evaluated (Figure 4d). As the current density increases from 0.1 to 0.2, 0.5, 1.0, 2.0 and 5.0 A·g^−1^, the different discharged capacities of 720, 650, 554, 479, 402 and 300 mAh·g^−1^ are delivered, respectively. The reversible capacities of the WS_2_/rGO electrode with 199 and 108 mAh·g^−1^ still can be achieved while the current density increases to 10.0 and 20.0 A·g^−1^, respectively. Significantly, after returning to 1.0 A·g^−1^, the capacity of WS_2_/rGO composites immediately recovered to 508.5 mAh^−1^, demonstrating the excellent electrode conductivity and fast Li^+^ diffusion. 

For in-depth reveal the role of rGO in the WS_2_/rGO electrode, the circulation property of the bare WS_2_ electrode has been assessed. The capacity of bare WS_2_ is only ~200 mAh·g^−1^ in Figure 4e, far lower than that of the WS_2_/rGO composites. In addition, the bare WS_2_ electrode suffers a sustaining capacity fading after the first few cycles, manifesting the prominent cycling property of the WS_2_/rGO on the side. The result is consistent with the conclusion of the morphology of the two materials in Figure 1a and Figure 1b. The rGO is formed stable seaweed-liked composite with WS_2_, avoiding a structure collapse of the electrode owing to the huge volume expansion and providing a highly conductive path for electron transport. Besides, the seaweed-liked structure materials will provide much more active sites, ameliorate the stresses arisen from volume variations and increase charge transfer rate to further improve lithium storage capacity and ionic conductivity [48].

**Figure 5:**
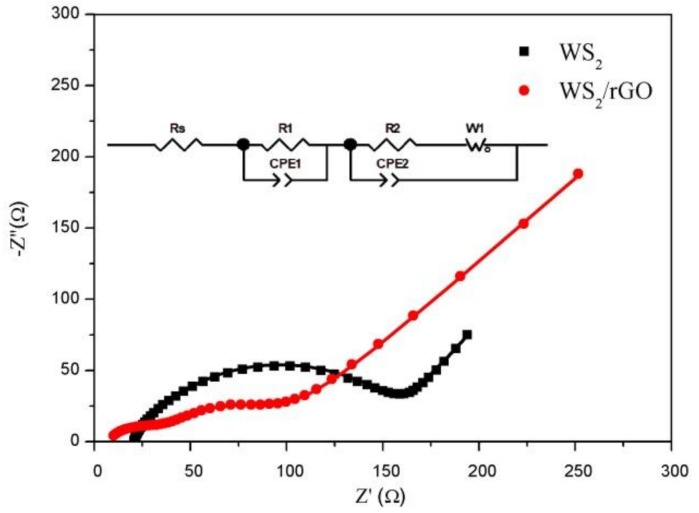
The Nyquist plots of bare WS_2_ and WS_2_/rGO composite range from 10^5^ Hz to 10^−2^ Hz after 100 cycles.

An EIS measurement was conducted for two samples to further research the electrochemical behavior in Figure 5. The tests in this survey were achieved after 100 cycles. Generally, the impedance spectra was composed of semicircle and a skew line, which were related to the charge transfer resistance and ion diffusion process, respectively [46]. It can be seen that the semicircle size of the WS_2_/rGO is much smaller than that of the bare WS_2_. The Rs and Rc of the bare WS_2_ and the WS_2_/rGO composites can be extracted by using equivalent circuit shown in the inset of Figure 5, which are 20.9, 116.4, 7.2 and 27.5 Ω, respectively, suggesting the faster charge transfer speed of WS_2_/rGO. Therefore, the addition of rGO can effectively enhance the conductivity performance of WS_2_ and greatly decrease charge transfer resistance, which is consistent with the expectation.

## 4. Conclusions

In summary, the seaweed-liked WS_2_/rGO nanocomposites was fabricated by a procedure involving the self-assembling of WO_4_^2−^, CTA^+^ with GO and the subsequent thermal treatment. The WS_2_/rGO material as the anode material for LIBs exhibited ultralong cycling life and striking rate performance with a stable and remarkable capacity of 507.7 mAh·g^−1^ at 1.0 A·g^−1^ after 1000 cycles. Furthermore, it was worth nothing that its capacities of 108 mAh·g^−1^ could still be maintained while the current density increases to 20.0 A·g^−1^. Noteworthily, after returning to 1.0 A·g^−1^, the capacity of WS_2_/rGO composites immediately recovered to 508.5 mAh^−1^. The superior electrochemical property could depend on the synergy of WO_4_^2−^, CTA^+^ and GO. The added GO is reduced to rGO during the calcining phase and formed stable seaweed-liked composite with WS_2_, not only providing an efficient conductive path for electron transport, but also avoiding a structure collapse of the electrode owing to the huge volume expansion. Moreover, the seaweed-liked structure would provide much more active sites, ameliorate the stresses arisen from volume variations and increase charge transfer rate to further improve lithium storage capacity and ionic conductivity. The seaweed-liked WS_2_/rGO nanocomposites show great promise for other energy storage devices (e.g., sodium-ion battery).
